# Interplay between Exonic Splicing Enhancers, mRNA Processing, and mRNA Surveillance in the Dystrophic *Mdx* Mouse

**DOI:** 10.1371/journal.pone.0000427

**Published:** 2007-05-09

**Authors:** Massimo Buvoli, Ada Buvoli, Leslie A. Leinwand

**Affiliations:** Department of Molecular, Cellular and Developmental Biology, University of Colorado at Boulder, Boulder, Colorado, United States of America; Max Planck Institute for Evolutionary Anthropology, Germany

## Abstract

**Background:**

Pre-mRNA splicing, the removal of introns from RNA, takes place within the spliceosome, a macromolecular complex composed of five small nuclear RNAs and a large number of associated proteins. Spliceosome assembly is modulated by the 5′ and 3′ splice site consensus sequences situated at the ends of each intron, as well as by exonic and intronic splicing enhancers/silencers recognized by SR and hnRNP proteins. Nonsense mutations introducing a premature termination codon (PTC) often result in the activation of cellular quality control systems that reduce mRNA levels or alter the mRNA splicing pattern. The *mdx* mouse, a commonly used genetic model for Duchenne muscular dystrophy (DMD), lacks dystrophin by virtue of a premature termination codon (PTC) in exon 23 that also severely reduces the level of dystrophin mRNA. However, the effect of the mutation on dystrophin RNA processing has not yet been described.

**Methodology/Principal Finding:**

Using combinations of different biochemical and cellular assays, we found that the *mdx* mutation partially disrupts a multisite exonic splicing enhancer (ESE) that is recognized by a 40 kDa SR protein. In spite of the presence of an inefficient intron 22 3′ splice site containing the rare GAG triplet, the *mdx* mutation does not activate nonsense-associated altered splicing (NAS), but induces exclusively nonsense-mediated mRNA decay (NMD). Functional binding sites for SR proteins were also identified in exon 22 and 24, and *in vitro* experiments show that SR proteins can mediate direct association between exon 22, 23, and 24.

**Conclusions/Significance:**

Our findings highlight the complex crosstalk between trans-acting factors, cis-elements and the RNA surveillance machinery occurring during dystrophin mRNA processing. Moreover, they suggest that dystrophin exon–exon interactions could play an important role in preventing *mdx* exon 23 skipping, as well as in facilitating the pairing of committed splice sites.

## Introduction

Dystrophin is a 427 kDa structural protein that connects the actin cytoskeleton with the basal lamina and protects the myofibers from mechanical stress produced during muscle contraction [1], [2]. Mutations in the very large dystrophin gene (2.4 million bases) are associated with both Duchenne muscular dystrophy (DMD), characterized by severe and progressive muscle degeneration, and the milder Becker muscular dystrophy (BMD). Dystrophin gene expression is controlled by several independent promoters, alternative splicing, and the use of different polyA^+^ signals. Although the gene covers about 2.4 megabases on the short arm of the X chromosome [3], [4], the 79 exons only encode a 14 kb mRNA [5]. Thus, 99.4% of hnRNA corresponds to intervening sequences that have to be removed during the splicing reaction. Pre-mRNA splicing consists of two consecutive trans-esterification reactions, and is carried out by the spliceosome, an RNA-protein complex composed of five small ribonucleoparticles called snRNPs and a large number of non-snRNP splicing factors [6], [7].

Splice site selection occurs by precise identification of poorly conserved consensus sequences located at the intron-exon boundaries. Commitment for a given set of splice sites appears to be enhanced by protein cross-talk across each exon, a process called exon definition [8], and by repression of numerous pseudosplice sites that are usually present in primary transcripts [9]. Specific cis-elements located in the exons, known as exonic splicing enhancers (ESEs), promote both constitutive and regulated splicing [10] and appear to be involved in exon definition [11]. Although ESEs were initially identified in alternative spliced exons as purine-rich regions [12]–[15], different motifs have been identified by different iterative selection strategies [16]–[19].

ESEs are recognized by SR proteins, a group of highly conserved modular splicing factors containing a N-terminal RNA-recognition domain (RRM), that mediates RNA binding, and a C-terminal arginine/serine rich domain, required for protein-protein interactions [20]. When bound to ESEs, SR proteins recruit and facilitate the binding of different components of the spliceosome to the nearby intron [10], [21], [22]. Moreover, they promote spliceosome assembly by directly contacting the pre-mRNA [23]–[25], as well as splice site selection by bringing exons into proximity [26], [27].

Even though the majority of ESEs have been found in enhancer-dependent introns containing a weak splice site, their discovery in constitutive exons [28]–[33], together with the finding that protein-coding sequences are enriched for potential SR binding sites [34]–[37], suggests that they could be present in the majority of exons. Fortuitous disruption of ESEs by missense, silent, and nonsense mutations, promotes exon skipping, intron retention, or exon size reduction. This process is different from the controversial nonsense–associated altered splicing (NAS), a post-transcriptional control pathway that detects disruptions in the open reading frame of precursor mRNAs. By regulating the synthesis of alternatively spliced isoforms skipping the nonsense mutation, NAS could prevent the production of deleterious truncated proteins [38]–[43]. While NAS affects only the splicing process, nonsense-mediated mRNA decay (NMD), the primary cellular response to mRNA carrying a premature termination codon (PTC), rapidly degrades fully spliced transcripts. NMD is activated during the first round of translation by PTCs placed at>50–55 nucleotides from the last exon–exon junction that is marked, after splicing, with an exon junction complex (EJC) that interacts with the termination factors [44]–[46].

The *mdx* mouse, the most widely used animal model for Duchenne muscular dystrophy, carries an ochre nonsense mutation in dystrophin exon 23 [47] that drastically reduces the abundance of dystrophin mRNA and consequently abolishes dystrophin expression [48]. Interestingly, a rare G residue precedes the terminal dinucleotide AG of intron 22, a configuration that is also present in the wild type strain. *In vitro*, GAG does not appear to function efficiently for the second step of splicing, and CAG to GAG mutations often result in exon skipping *in vivo* [49]–[54]. These findings explain the very low presence of GAG trinucleotides at natural 3′ splice sites [55].

A promising RNA “re-framing” strategy based on antisense oligonucleotides that promote skipping of exons carrying nonsense mutations has recently been developed and tested in the *mdx* mouse system [56]. However, the potential impact of the *mdx* mutation on dystrophin mRNA processing has not yet been examined. In this study we investigated the molecular events triggered by the *mdx* mutation on mRNA metabolism, the functional activity of the GAG present in intron 22, and the potential relevance of SR proteins on dystrophin mRNA splicing.

## Results

### Identification of dystrophin exon 23 splicing enhancers

**Figure 1:**
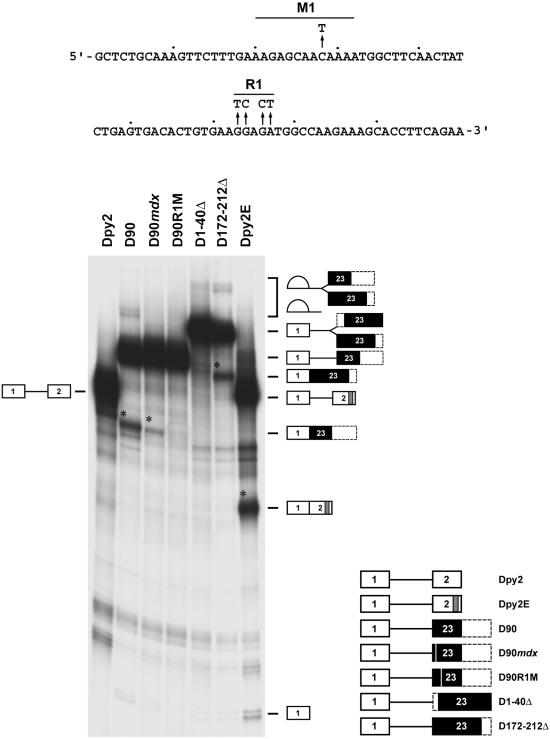
Exon 23 contains purine-rich elements that activate splicing of a heterologous substrate containing a weak 3′ splice site. The sequence of the first 90 nucleotides of dystrophin exon 23 is shown at the top of the figure. Arrows identify the natural mutation (C→T) occurring in the *mdx* mouse and the mutations that disrupt the purine-rich region R1. The borders of the M1 element are defined by the homology (12 of 12 positions) shared with the human dystrophin exon 23. Different regions of dystrohin exon 23 were inserted in the second exon of Dpy2, an enhancer-dependent mRNA containing a weak pyrimidine stretch [60]. The structure of the chimeric pre-mRNA substrates subjected to *in vitro* splicing reactions is shown at the bottom right of the figure. White boxes indicate Dpy2 exons, black boxes indicate different regions of the dystrophin exon 23, dashed boxes indicate portions of exon 23 not included in the substrates, white vertical lines indicate the *mdx* and R1 mutations. The gray box in Dpy2E identifies the two splicing enhancers found in the α-tropomyosin exon 2 gene [60]. The structures and the mobility of the products and intermediates of splicing are shown on the sides of the gel. D172-212Δ RNA was used as a positive control for D1-40Δ to rule out any exon size inhibitory effect on splicing. Asterisks indicate the position of the spliced RNAs. Splicing reactions, carried out for 70 min at 30°C, were resolved on a 12% denaturing polyacrylamide gel. Comparable results were obtained in three independent experiments.

Since it has been reported that ESEs are present in several dystrophin exons and their inactivation can cause perturbations in the gene splicing pattern [28], [57]–[59], we began our analysis by determining whether exon 23 contains ESEs. To this aim, different regions of the exon 23 were cloned into the enhancer-dependent Dpy2 pre-mRNA [60], an heterologous construct that carries a weak polypyrimidine tract and does not splice *in vitro* (Figure 1, Dpy2). A minimal region, capable of activating Dpy2 splicing *in vitro,* was mapped to the first 90 nucleotides of exon 23 (Figure 1, D90). The *mdx* C→T transversion located in a 12 nucleotides conserved region (M1element), did not disrupt any of the potential high-score SR motifs for SF2/ASF, SC35, SRp40 and SRp55 [61], [62] but reduced D90 splicing more than 40% (Figure 1, D90*mdx*). Mutagenesis of a purine-rich region (R1 element) matching a splicing enhancer previously identified by *in vitro* selection [16], [18], completely abolished splicing activation (Figure 1, D90R1M) that was not restored by downstream exonic sequences (data not shown). To define further the ESE, we determined that the first 40 nucleotides of exon 23 containing only M1, did not activate Dpy2 splicing (data not shown). However, their deletion abolished Dpy2 activation (Figure 1, D1-40Δ) suggesting that the exon 23 ESE is a multipartite enhancer consisting of two elements, M1 and R1. Finally, when compared with the well-characterized α-tropomyosin exon 2 enhancer elements (Dpy2E construct), exon 23 ESE stimulated Dpy2 splicing less efficiently (2.5 fold reduction).

These data indicate that the constitutive dystrophin exon 23 contains a multisite-ESE that is partially inactivated by the *mdx* nonsense mutation. Thus, reduction of dystrophin mRNA levels observed in the *mdx* mouse could result from aberrant splicing caused by the disruption of this splicing cis-element.

### SR proteins interact with dystrophin exon 23 splicing enhancer

**Figure 2:**
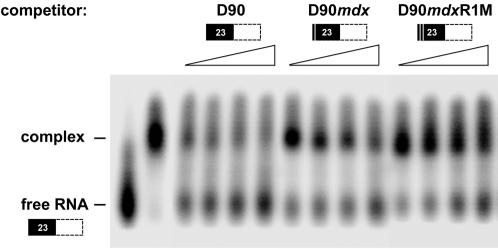
SR proteins bind exon 23 enhancer elements. ^32^P labeled D90 RNA was incubated at 30°C for 10 min with purified SR proteins prepared from calf thymus as previously described [63]. Binding reactions performed in the absence or in the presence of increasing concentrations of cold competitor RNAs (with molar excess of 30, 60, 90 and 120 fold, as indicated by the gradients above the gel lanes) were fractionated on a 4% non-denaturing polyacrylamide gel. The schematic representation of the RNAs is depicted as in Figure 1. The positions of free RNA and bound complexes are shown. Comparable results were obtained in two independent experiments.

**S**R proteins bound to ESEs promote spliceosome assembly and pre-mRNA processing. To determine whether SR proteins could form a stable complex with the exon 23 ESE, an RNA mobility shift assay was performed. As shown in Figure 2, a retarded RNA-protein complex was resolved on a native gel following incubation of ^32^P-labeled D90 RNA and calf thymus purified SR proteins (second lane from left). An excess of D90 cold competitor successfully prevented the efficient formation of the complex, whereas the competitor carrying the *mdx* mutation (D90*mdx*) was much less effective. In contrast, the double mutant D90*mdx*R1M was unable to significantly inhibit complex formation even at the highest concentration of competitor tested. Thus, SR proteins specifically bind exon 23 ESE and could mediate the enhancing activity of D90 observed in the heterologous Dpy2 construct. As judged by the effect of the D90*mdx* competitor on complex formation, it appears that the *mdx* mutation interferes with SR assembly on exon 23 ESE.

**Figure 3:**
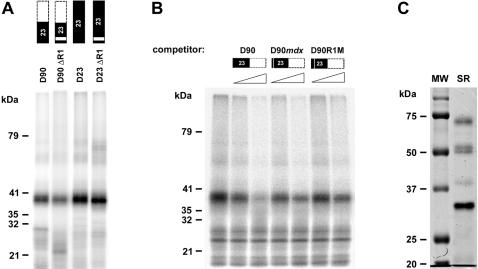
UV cross-linking of a 40 kDa SR protein to exon 23. A) Different ^32^P labeled RNAs represented as in Figure 1 were incubated at 30°C for 10 min with purified SR proteins. Following UV cross-linking and RNAse A/T1 digestion, ^32^P labeled proteins were analyzed by SDS-PAGE. Molecular weight markers are shown on the left. B) Cross-linking of ^32^P labeled D90 RNA was performed in the presence of increasing amounts of cold competitor RNAs (10 and 30 fold molar excess, as indicated by the gradients above the gel lanes). C) Coomassie–stained SDS-PAGE separated SR proteins (7 µg). Comparable results were obtained in three independent experiments.

UV cross-linking experiments were then carried out to determine which SR protein(s) might directly contact the M1 and/or the R1 elements. Uniformly ^32^P-labeled D90 RNA was incubated with purified SR proteins, UV irradiated and RNase digested as described in the Materials and Methods section. The resulting radioactive complexes were then resolved by SDS-PAGE. As shown in Figure 3A, a 40 kDa SR protein was efficiently cross-linked to the first 90 nucleotides of exon 23 containing both M1 and R1 (D90). A reduction in complex formation was observed after deletion of R1 (D90ΔR1), confirming the functional relationship between M1 and R1 previously observed in the *in vitro* splicing reaction. A 40 kDa protein was also the only polypeptide cross-linked to the whole exon 23 RNA (D23). Nevertheless, the comparison between the intensity of the D90ΔR1 and D23ΔR1 signals (with a difference of about 2.5 fold) strongly suggests that another 40 kDa protein binding site is located downstream of D90. Binding specificity for the M1 and R1 sites was then demonstrated by the addition to the reactions of the mutant cold competitors D90*mdx* and D90R1M. As shown in Figure 3B, both RNAs had a similar effect in reducing the 40 kDa cross-linking when compared with the wild type D90. Selective enrichment of the 40 kDa protein in our SR preparation was ruled out by Coomassie-stained SDS-PAGE gel that showed the expected SR protein relative ratio (Figure 3C, SR lane) [63]. Thus, the natural *mdx* mutation could impair splicing efficiency by reducing the binding of a 40 kDa SR protein to exon 23 ESE.

### *In vivo* analysis of mutations affecting exon 23 ESE activity

**Figure 4:**
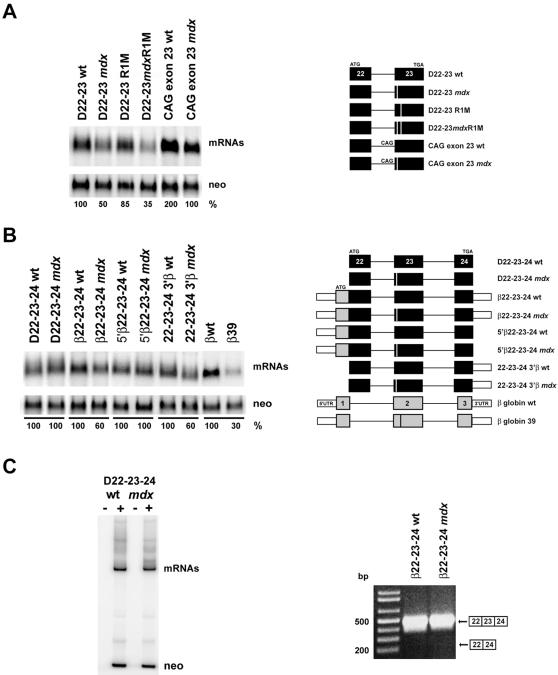
Splicing of dystrophin mini-genes *in vivo.* Cos 7 cells were transfected with plasmids expressing different portions of the genomic region of the dystrophin gene spanning from exon 22 to exon 23/24. Northern blot analysis was carried out on total RNA prepared 24 hr after transfection, as described in Materials and Methods. Band intensities, quantified by phosphorimager analysis, were normalized for transfection efficiency and RNA recovery to the level of the co-expressed neomycin mRNA (neo). Normalized values were expressed as a percentage of wt mRNAs that were defined as 100. The schematic structure of the substrates is shown on the right part of both panels A and B. Dystrophin exons are identified as in Figure 1. β-globin exons are shown as gray boxes while the 5′ and 3′ UTRs as white thinner boxes. (A) Two exon mini-genes carrying dystrophin exons 22 and 23. (B) Three exon mini-genes carrying dystrophin exon 22 and 23 and 24, chimeric constructs, and the β-globin system (β−globin wt and β−globin 39 carrying a nonsense mutation at codon 39) used as a reference [81]. Comparable results were obtained in three independent experiments. (C) Left panel: autoradiogram of a representative quantitative RT-PCR performed as described in Materials and Methods. The upper bands correspond to RT-PCR products from dystrophin mRNA obtained with primers located in exons 22 and 24; the lower bands correspond to the neomycin gene used as an internal control. −and+lanes correspond to reactions carried out in absence or presence of reverse transcriptase. Right panel: non-quantitative RT-PCR performed as described in Materials and Methods. Mobility of the predicted splicing products is shown on the right. The PCR product deriving from skipping of exon 23 was not detected.

The relevance of the *mdx* and R1 mutations and their potential effects on dystrophin mRNA metabolism were next studied in cell culture. COS 7 cells were transfected with model substrates carrying different portions of the dystrophin gene. Two-exon constructs, containing the genomic sequence spanning from exon 22 to exon 23, were utilized to study the effect of the mutations on mRNA splicing. Three-exon constructs, containing the genomic sequence spanning from exon 22 to exon 24 and therefore sensitive to mRNA surveillance, were employed to examine both NAS and NMD activities. Translation start and termination codons were inserted in frame with the dystrophin exons at the beginning of exon 22 and at the end of exon 23 or 24 respectively. Twenty-four hours after transfection, total RNA was isolated and quantitative analyses were carried out on Northern blots normalized to the internal control neomycin (see Materials and Methods). As shown in Figure 4A, the *mdx* mutation decreased the levels of RNA to ∼50% (D22-23 *mdx*), while the R1 mutation (D22-23 R1M) had a milder phenotype than predicted from the *in vitro* splicing experiments (Figure 1, D90R1M), reducing the processing of the D22-23 R1M RNA to ∼85%. The observation that the simultaneous presence of the two mutations had an additive negative effect on splicing (D22-23 *mdx*R1M construct, reduction to ∼35%) probably reflect the previous finding that only one splicing enhancer complex can interact with the spliceosome components at any given time [64], [65].

Since it has been shown that the trinucleotide GAG severely slows down the second step of splicing *in vitro* [49], [52], and in different human genetic diseases is associated with aberrant splicing phenotypes [51], [53], [54], we next investigated if the presence of the ESE in the constitutive exon 23 is required to activate the 3′ splice site of intron 22 containing the rare G residue at position −3. Mutant mini-genes carrying the canonical CAG trinucleotide at the 3′ splice site of intron 22 were generated and their activity examined. As shown in Figure 4A, splicing increased about 2 fold for both the wild-type and the *mdx* RNA (CAG exon 23 wt, CAG exon 23 *mdx*), but their relative abundance perfectly matched the ratio observed in the GAG constructs (∼50% reduction for the *mdx* RNA). The possibility that intron 23 5′ splice site could enhance GAG recognition [66] was ruled out by the analysis of the three-exon constructs carrying the trinucleotide CAG. In this case, splicing stimulation, as well as comparative RNA ratios, perfectly matched the results previously obtained with the two-exon constructs (data not shown). Thus, as opposed to other more specialized ESEs involved in the activation of non-canonical 3′ splice sites [67], exon 23 ESE appears to be a constitutive element, not functionally dedicated to activate the GAG located at the 3′ splice site of intron 22.

Surprisingly, the presence of the *mdx* mutation had essentially no effect on RNA abundance of the three-exon constructs as determined by both Northern blot (Figure 4B D22-23-24 wt, D22-23-24 *mdx*) and quantitative RT-PCR (Figure 4C left panel). In addition, no levels of exon 23 skipped RNA were detected when the radioactive PCR was carried out for 34 cycles (data not shown). Moreover, Western blot analysis revealed that exon 22-23-24 mRNA was competent for translation, producing a polypeptide of about 20 kDa (data not shown). The discovery that the levels of expression of the three-exon mini-genes (comparable, after normalization, to the two exon constructs) were about 4-5 fold lower than those of the β-globin constructs (Figure 4B, compare D22-23-24 wt with βwt), prompted us to investigate if mRNA instability was interfering with NMD detection. To address this issue, we built new hybrid constructs fused to the β-globin 3′ UTR, that carries stabilizing cis-elements recognized by cytoplasmatic RNP complexes [68]. To boost the efficiency of translation we also linked the β-globin 5′UTR and exon 1 to dystrophin exon 22 [69]. Examination of these constructs revealed a 3–4 fold increase in RNA levels, with a significant reduction in the abundance of the mRNA carrying the *mdx* mutation to ∼60% (Figure 4B, β22-23-24 wt and β22-23-24 *mdx*). Interestingly, similar results were obtained in the presence of the β-globin 3′UTR alone (22-23-24 3′β constructs), while no effect on dystrophin mRNA metabolism was detected in constructs carrying the β-globin 5′UTR alone (Figure 4B, 5′β22-23-24wt and *mdx*). These results, together with the finding that NAS activity was not detectable in the β-globin hybrid constructs either (Figure 4C, right panel), support the hypothesis that reduction of dystrophin mRNA observed *in vivo* is probably due to NMD.

The finding that the D22-23-24, but not the D22-23 mini-genes constructs, were insensitive to the presence of the *mdx* mutation, also indicates that the presence of intron 23 and exon 24 compensate for the partial inactivation of exon 23 ESE. The hypothesis that splicing between exons 23 and 24 could compensate for the *mdx* mutation by creating a new ESE [70] was then examinated by testing RNA substrates containing juxtaposed exons 23 and 24. Although RNA abundance was still reduced by the *mdx* mutation, only a mild compensatory effect (∼10–15%) was observed with these constructs (data not shown).

### SR proteins promote dystrophin exon bridging *in vitro*

In addition to their involvement in the splicing reaction, SR proteins and ESEs appear to mediate exon/exon association, a process that, bringing exons into proximity, could facilitate selection of the correct 5′ and 3′ splice sites [26], [27]. In a gene like dystrophin, characterized by large introns, exon-bridging could play an important role before mRNA catalysis. Moreover, if not affected by the *mdx* mutation, associations between exon 22, 23 and 24, could force the inclusion of exon 23 and prevent its skipping.

**Figure 5:**
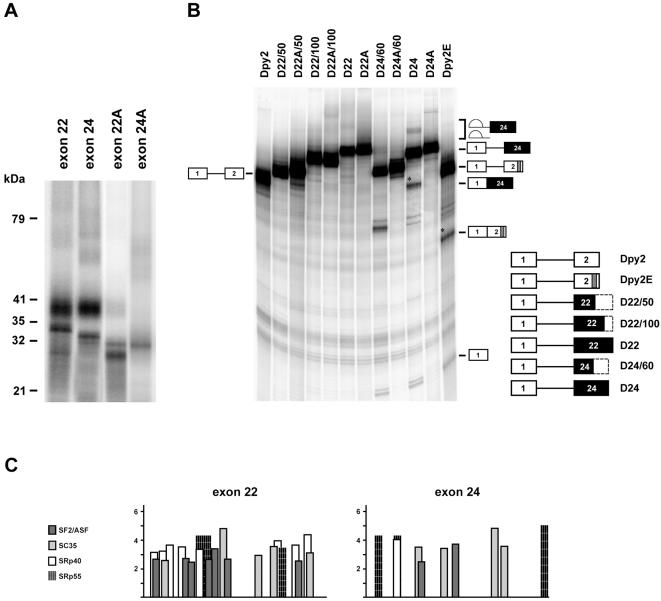
SR proteins interact with dystrophin exon 22 and 24. A) Different SR proteins associate with exons 22 and 24. ^32^P labeled exon 22 and 24 RNAs, incubated with SR proteins were subjected to UV-crosslinking as described in Figure 3. Molecular weight markers are shown at the left; exon 22A and 24A correspond to the antisense RNAs used as controls. B) Exon 24, but not exon 22 activates splicing of the enhancer-dependent Dpy2 RNA. Different regions of dystrohin exon 22 (145 nt) and 24 (113 nt) were cloned in the second exon of the enhancer-dependent Dpy2 substrate. The structure of the chimeric constructs subjected to *in vitro* splicing reactions is shown on the right of the panel, with black boxes indicating exon 22 and 24 and dashed boxes the portion of the corresponding exon not included in the pre-mRNA. A indicates the anti-sense orientation of each region, while numbers after the slash indicate their nucleotide length. Dpy2 and Dpy2E are defined as in Figure 1. The structures and the mobility of the products and intermediates of splicing are shown on both sides of the panel. Asterisks indicate the position of the spliced RNAs. The molecular weigh of the band detected below the D24/60 RNA precursor does not correspond to the expected splicing product. *In vitro* splicing reactions were carried out as in Figure 1. Comparable results were obtained in two independent experiments. C) Schematic representation of SR binding sites within dystrophin exon 22 and 24 computed by ESEfinder [61], [62].

To determine whether SR proteins interact with the constitutive exons 22 and 24, we performed UV crosslinking experiments with purified SR proteins, uniformly ^32^P-labeled exon 22 and 24 RNAs and nonspecific cold competitor RNA,. This analysis revealed that, in addition to a 40 kDa protein(s), exons 22 and 24 efficiently interacted with two other SR proteins, migrating at ∼34 kDa (Figure 5A). Neither of these SR proteins cross-linked to the antisense-exons used as controls for binding specificity (exon 22A and exon 24A). Of the known SR proteins, ASF/SF2, SC35 and 9G8 migrate as 34 kDa peptides.

To determine whether SR proteins bound to the constitutive exons 22 and 24 could also activate splicing in an enhancer-dependent context, we cloned different regions of the two exons in the Dpy2 pre-mRNA. As shown in Figure 5B, only the entire exon 24 was able to stimulate Dpy2 splicing quite efficiently. However, it is important to keep in mind that the strength of each ESE is determined by different parameters [65]. Thus, association of particular SR proteins with constitutive exons can result in the formation of complexes involved only in the basic splicing reaction and not having enough potency for activating weak splice sites (see Discussion).

**Figure 6:**
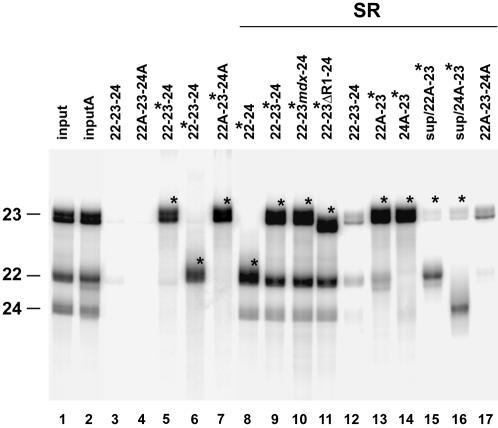
SR proteins mediate associations of exons 22, 23 and 24. Biotinylated and non-biotinylated dystrophin exons labeled with ^32^P-ATP (sense exons) or ^32^P-UTP (antisense exons), were mixed with purified SR proteins. Following incubation, biotinylated RNAs were purified with streptavidin magnetic beads and the formation of RNA complexes was analyzed on 10% denaturing polyacrylamide gel. Asterisks and A indicate biotinylated and antisense exons respectively. Lanes 1 and 2, showing the relative migration of exons 22, 23 and 24, contain one-fourth of the RNA inputs assayed in lanes 8–11 and 13–14 respectively. Lane 15 and 16 contain one-fourth of the supernatants collected from reactions loaded in lanes 13–14. Background levels, probably generated by non-specific trapping of RNA or RNA/protein complexes into the magnetic beads, are shown in lanes 3, 4, and 12 and 17 respectively. Comparable results were obtained in three independent experiments.

We next asked whether SR proteins and ESEs could directly promote dystrophin exon bridging *in vitro*. Biotinylated ^32^P-labeled exon RNAs were incubated with non-biotinylated ^32^P-labeled exon RNAs and purified SR proteins diluted to the same concentration measured in splicing HeLa nuclear extract (∼30 µg/ml) [71]. Possible ternary complexes (biotinylated exon/SR proteins/non-biotinylated exon) were purified with streptavidin magnetic beads and, after extensive washing, RNA was extracted and resolved on denaturing gels. This assay revealed that exon/exon associations were efficiently promoted in the presence of SR proteins. In fact, a substantial enrichment of co-purified non-biotinylated exons (∼10 fold over the experimental background), was consistently detected after addition of SR proteins to the reactions (Figure 6, compare lanes 8–11 with lane 12). However, neither the presence of the *mdx* mutation nor the deletion of the R1 element affected the efficient formation of complexes between exon 22, 24 and biotinylated exon 23 (Figure 6, compare lanes 10 and 11 with lane 9). The specific formation of ternary complexes is demonstrated by the findings that (i) in the absence of SR proteins only biotinylated RNAs were purified (Figure 6, lanes 5–7) and (ii) antisense exons 22 or 24, used as negative controls and whose stability was not influenced by the presence of the SR proteins (Figure 6, lanes 13 and 14), did not associate with biotinylated exon 23. Thus, the bridging activity of SR proteins, that in not impaired by the *mdx* mutation, could be involved in counterweighing exon 23 skipping predisposition caused by the combination of the mutation and the unusual trinucleotide GAG.

## Discussion

The physical characteristics of the dystrophin gene provide a unique model for expanding our knowledge of the mechanisms and factors involved in splice site selection and RNA processing. Dystrophin introns, some of which span more than 100 kb, appear to be spliced during the 16 hours required to transcribe the entire gene [72]. This coordinate activity probably provides an efficient way to increase the local concentration of splicing factors nearby the hnRNA, reduces the number of splice sites available and ensures that exons are spliced in the correct 5′ to 3′ order [73]. However, little is known about the factors and the cis-elements involved in dystrophin mRNA splicing.

In this study, we report that in the *mdx* mouse the presence of a nonsense mutation in exon 23 partially inactivates a multisite exonic splicing enhancer. Although this event could cause exon skipping, we found that splicing occurs normally and the mRNA carrying the nonsense mutation is degraded by activation of the NMD pathway. Here we provide evidence that exon 23 contains multiple binding sites for a 40 kDa SR protein. The M1 element is highly conserved between both human and mouse dystrophin genes (12 out of 12 positions), while R1 matches a splicing enhancer that was previously identified by *in vitro* functional iterative strategies [16], [18]. Several SR binding sites having enhancing activity have also been identified and studied in the constitutive β-globin exon 2 [29], and other observations have demonstrated a linear correlation between splicing efficiency and the number of enhancer elements present [65]. Our data are in agreement with this model and also show the functional flexibility of adjacent ESEs. In fact, while both M1 and R1 were necessary to activate splicing of the enhancer dependent Dpy2 construct, in the context of the dystrophin gene, intron 22 removal is mainly dependent on the activity of M1. Nonetheless, in the presence of the *mdx* mutation, R1 could play an important role as a back-up element, which partially compensates for the lack of M1 activity. By UV cross-linking and band shift experiments, we have shown that both M1 and R1 can be specifically recognized by a 40 kDa SR. Although both Tra 2α/β and SRp40 migrate at ∼40 kDa, Tra 2 is not present in purified preparations of SR proteins [74], therefore SRp40 should be the only protein that binds exon 23 ESE.

In this paper we also show that exon 23 ESE activity is not functionally linked to the rare GAG present at the 3′ splice site of intron 22. Two lines of evidence support this conclusion: i) two exon mini-genes carrying the most efficient CAG were still equally sensitive to the *mdx* mutation, and ii) in the human dystrophin gene M1 is conserved, while the GAG is replaced by CAG. By quantitative RT-PCR employing different exon/intron primers [75], we found that splicing of a mini-gene carrying exons 22, 23, and 24 did not follow the first-come, first-served model. Nevertheless, intron 23 was spliced before intron 22 in only 50% of the molecules. At an RNA polymerase average elongation rate of 2.4 kb min^−1^ [72], intron 22 3′ splice site becomes available for splicing just a minute before intron 23 3′ splice site synthesis. Therefore, we believe that lack of linear progression in intron removal is not caused by a severe delay promoted by the GAG. Our mini-gene analysis also rules out the possibility that the 16 hours necessary to transcribe a gene as large as dystrophin could provide enough time for intron 22 removal, even with its slow kinetic of cleavage. Our results indicate that in vivo, removal of dystrophin intron 22 is not subjected to the same functional limitations observed previously in vitro [49], and support the hypothesis that the use of natural GAG 3′ splice sites could be forced by the absence of downstream competing CAGs [52], as in the case of dystrophin exon 23 (data not shown). The lack of cross-talk between ESEs and GAG could explain why just a small minority of natural 3′ splice sites contain a GAG while ESEs appear to be present in most exons.

Here we also show that the SR proteins can directly promote *in vitro* exon/exon association between the constitutive dystrophin exons 22, 23 and 24. Alternative splicing of the chicken cardiac troponin T (cTnT) gene appears to be modulated through exon/exon interactions mediated by SR proteins [27], and ESEs are required for mammalian trans-splicing [76]. Our data show that *in vitro*, the formation of a ternary complex between SR proteins and different dystrophin exons occurs efficiently and is not influenced by the presence of the *mdx* mutation. Interactions of exon 22, 23 and 24 mediated by SR proteins, could hold the dystrophin pre-mRNA in a topological configuration that imposes the use of intron 22 3′ splice site. Pre-splicing physical interactions between exon 23 and 24 could increase the local concentration of ESEs around intron 22 3′ splice site [65] and compensate for the damaging effects of the *mdx* mutation. Thus, the splicing stimulation observed with the three-exon constructs (Figure 4A) could derive from the combined action of the ESEs contained in the two unspliced exons.

In the dystrophin gene, selection of committed splice sites often separated by long distances (>100 kb) could be achieved by intron looping promoted by redundant ESEs situated in constitutive exons that are not functionally subordinated to splice sites strength or distance. Interestingly, this mechanism has been also proposed for several hnRNP proteins bound to intronic sequences [77]–[79]. The finding that purine rich regions with a wide spectrum of enhancing activity have been identified in several other dystrophin exons [31] indicates that ESEs could be common elements along the gene and could share the same bridging properties of the ones we have analyzed. Thus, optimization of therapeutic antisense oligonucleotides promoting exon skipping and preventing the incorporation of nonsense mutations in the mature dystrophin RNA should take into account both the complex crosstalk between ESEs, SR and pre-mRNA and potential exon/exon associations.

## Materials and Methods

### DNA constructs

The dystrophin genomic region spanning exons 22, 23 and 24 was amplified from wild type and *mdx* genomic DNAs with ExpandTM Long template PCR system (Roche) and cloned in the *EcoR*V site of both pcDNA3.1(+) and pcDNA3.1/Myc-His(−) A (*Invitrogen*). The translation start and termination codons were inserted in frame with the dystrophin exons at the beginning of exon 22 and at the end of exon 23 or 24 during the PCR reaction. Minigenes were fused to the 3′ UTR of bovine growth hormone carried by the cloning vectors. Single dystrophin exons were cloned into the *Pst*I site of pUC18-Dpy2 [60], generating Dpy2/22, Dpy2/23wt, Dpy2/23*mdx* and Dpy2/24 plasmids. Mutant constructs were prepared by inverse PCR [80] with appropriate oligonucleotide pairs. Templates for *in vitro* RNA transcription were generated by PCR using primers containing the T7 transcription initiation sequence in the 5′ oligonucleotides. Human β-globin reporter plasmids βwt and β39 were as described [81]. All oligonucleotide sequences and cloning strategies are available upon request.

### DNA transfection

COS 7 cells were grown in 100 mm dishes containing DMEM (Gibco-BRL) supplemented with 10% fetal bovine serum (Gibco-BRL). Transfections were carried out with 3 µg of each construct using Lipofectamine reagent (Gibco-BRL) according to the manufacturer's instructions. RNA was isolated 20 hr after transfection.

### RNA extraction, Northern Blot and RT-PCR

Total RNA was isolated from cultured cells using TRI REAGENT (Molecular Research Center, Inc.) according to the manufacturer's instructions. Northern Blot analysis was performed as previously described on 10 µg of total RNA [82]. Blots shown in Figure 4 were hybridized with oligonucleotide 24E complementary to dystrophin exon 24 (5′-TCTGCATTGTTTGAGCTGTTTTT-3′), and oligonucleotide 2glo complementary to β-globin exon 2 (5′-CACTAAAGGCACCGAGCACTTT-3′). Oligonucleotide 22E complementary to dystrophin exon 22 (5′-CTGTAATTTCCCGAGTCTCTCC-3′), and oligonucleotide 23E complementary to dystrophin exon 23 (5′-CTGAAATTTTCGAAG TTTATTC-3′) were also used to confirm the results obtained with the previous set of oligonucleotides. Oligonucleotide Neo3 (5′-AAGGTGAGATGACAGGAGATC-3′) was used to normalize the blots to the internal control neomycin. Band intensities were quantified by phosphorimager analysis (Storm 860 image analyzer, Molecular Dynamics). For RT-PCR analysis, total RNA was treated with DNase I Amp Grade (Gibco-BRL) and cDNA synthesized with random hexamers (Gibco BRL) and SUPERSCRIPT II reverse transcriptase (Gibco BRL) according to the manufacturer's instructions. For quantitative amplification (Figure 4C, left panel) PCR reactions were carried out in the presence of α ^32^P dCTP as previously described [83] with primers 22E, 22E5 (5′-TTTTTGACACTTTACCACC AAT-3′), 24E, Neo5 (5′-TGAATGAACTGCA GGACGAGG-3′) and Neo3. PCR reactions were analyzed on 8% acrylamide gels after 10, 15, 20 35 cycles and bands quantified by phosphorimager analysis. The non-quantitative PCR carried out to monitor exon 23 skipping (Figure 4C, right panel), was performed with oligonucleotides 22E and 24E (35 cycles). PCR reactions were assessed on a 1.2% agarose gel and stained with EtBr.

### *In vitro* transcription and splicing reactions

Capped ^32^P–labeled RNAs were synthesized with T7 RNA polymerase, as previously described [84]. Unlabelled competitor RNAs were prepared using the MEGAscript ™ T7 Kit (Ambion) following the manufacturer's instructions. Standard splicing reactions contained 20–40 fmol of ^32^P-labelled RNA transcript, 2.2 mM MgCl_2_, 0.5 mM ATP, 20 mM creatine phosphate, 1000 U/ml Rnasin (Promega) and 40% nuclear extract. Splicing products, fractionated on 12% polyacrylamide (acrylamide:bisacrylamide 20:1)/8M urea gels, were quantified by phoshorimager analysis.

### SR proteins

SR proteins were purified as previously described [63]. Gel-shift, UV cross-linking, and exon bridging assays were carried out with a final SR protein concentration of ∼30 µg/ml, corresponding to the one measured in HeLa splicing nuclear extracts [71].

### Gel-shift assay

Binding reactions for the gel-shift assays, containing purified SR proteins [63], 100 fmol RNA, 5 mg/ml heparin, 20 mM HEPES pH 8, 5% glycerol, 70 mM KCL and 3 mM MgCl_2_, were incubated at 30°C for 10 minutes. Cold competitor RNAs were pre-incubated with SR proteins for 5 minutes prior to the addition of radiolabeled substrates. Complexes were fractionated on 4% polyacrylamide gels (acrylamide/bisacrylamide 80:1), 5% glycerol, 0.5×TBE. Signals were quantified by phoshorimager analysis.

### UV cross-linking

UV cross-linking experiments were carried out as for the gel shift-assay, except that heparin was omitted from the reactions and RNA samples were labeled with ATP. After 10 minutes incubation at 30°C, reactions were transferred in a 96-well microtiter dish (Falcon Micro Test III™), placed directly under a 254-nm UV light at 5 cm from the bulb, and irradiated for 10 minutes. RNAse A (1mg/ml) and T1 (10U) were then added and the reactions were incubated for 15 minutes at 37°C. Samples, adjusted to 40 mM Tris (pH 6.8), 7% glycerol, 1.5% SDS and 400 mM 2-mercaptoethanol, were then fractionated on 12% SDS-PAGE gel. Signals were quantified by phoshorimager analysis.

### Exon bridging assay

Biotinylated RNAs were prepared as capped ^32^P–labeled RNAs, except that ATP or UTP were used as radiolabeled nucleotides for sense or antisense exons respectively and the concentrations of both CTP and Biotin-14-CTP (Gibco-BRL) were 250 µM. Before use, 0.6 mg of Streptavidin MagneSphere® Paramagnetic Particles (Promega) were both washed (3 times with 1ml) and resuspended (100 µl) in buffer W (20 mM HEPES, 100 KCL, 3 mM MgCl_2_, 5 µg/ml tRNA, and 20 µg/ml BSA). Exon/exon interactions were carried out in 10 µl reactions containing 20 mM HEPES, 100 KCL, 3 mM MgCl_2_, 20 units of RNasin (Promega), equimolar amounts of biotinylated and nonbiotinylated RNAs (200 fmoles each) and purified SR proteins (∼30 µg/ml). After incubation at 30°C for 10 minutes, streptavidin beads were added and reactions incubated at 4°C for 1 hour with nutation. Beads were then collected by magnetic stand (Promega), and while the aqueous fractions were phenolized and then ethanol precipitated, beads were washed 4 times with 1 ml of buffer W containing 0.05% Triton-X-100 and then resuspended in 100 µl of elution buffer (1% SDS, 1 mM EDTA, 50 µg/ml tRNA). Samples were incubated at 90°C for 5 minutes, beads collected and supernatants, containing the released RNAs, extracted with phenol/chloroform and ethanol precipitated. RNAs were analyzed on 10% polyacrylamide-8M urea gels. Signals were quantified by phoshorimager analysis.
